# Emergency Department Triage Scales and Their Components: A Systematic Review of the Scientific Evidence

**DOI:** 10.1186/1757-7241-19-42

**Published:** 2011-06-30

**Authors:** Nasim Farrohknia, Maaret Castrén, Anna Ehrenberg, Lars Lind, Sven Oredsson, Håkan Jonsson, Kjell Asplund, Katarina E Göransson

**Affiliations:** 1 The Swedish Council for Health Technology Assessment and Dep of Medical Sciences, Uppsala University Hospital, Uppsala, Sweden; 2 Dept of Clinical Science and Education and Section of Emergency Medicine, Södersjukhuset (Stockholm South General Hospital) Stockholm, Sweden; 3 School of Health and Social Studies, Dalarna University, Falun, Sweden; 4 Dept of Medicine, Uppsala University Hospital, Uppsala, Sweden; 5 Dept of Emergency Medicine, Helsingborg Hospital, Helsingborg, Sweden; 6 Dept of Orthopedics, Uppsala University Hospital, Uppsala, Sweden; 7 Dept of Public Health and Clinical Medicine, University Hospital, Umeå, Sweden; 8 Dept of Emergency Medicine, Karolinska University Hospital, Solna, Sweden; 9 Dept of Medicine, Karolinska Institutet, Solna, Sweden

## Abstract

Emergency department (ED) triage is used to identify patients' level of urgency and treat them based on their triage level. The global advancement of triage scales in the past two decades has generated considerable research on the validity and reliability of these scales. This systematic review aims to investigate the scientific evidence for published ED triage scales. The following questions are addressed:

1. Does assessment of individual vital signs or chief complaints affect mortality during the hospital stay or within 30 days after arrival at the ED?

2. What is the level of agreement between clinicians' triage decisions compared to each other or to a gold standard for each scale (*reliability*)?

3. How valid is each triage scale in predicting hospitalization and hospital mortality?

A systematic search of the international literature published from 1966 through March 31, 2009 explored the British Nursing Index, Business Source Premier, CINAHL, Cochrane Library, EMBASE, and PubMed. Inclusion was limited to controlled studies of adult patients (≥15 years) visiting EDs for somatic reasons. Outcome variables were death in ED or hospital and need for hospitalization (validity). *Methodological quality and clinical relevance *of each study were rated as high, medium, or low. The results from the studies that met the inclusion criteria and quality standards were synthesized applying the internationally developed GRADE system. Each conclusion was then assessed as having strong, moderately strong, limited, or insufficient scientific evidence. If studies were not available, this was also noted.

We found ED triage scales to be supported, at best, by *limited *and often *insufficient *evidence.

The ability of the individual vital signs included in the different scales to predict outcome is seldom, if at all, studied in the ED setting. The scientific evidence to assess interrater agreement (reliability) was limited for one triage scale and insufficient or lacking for all other scales. Two of the scales yielded limited scientific evidence, and one scale yielded insufficient evidence, on which to assess the risk of early death or hospitalization in patients assigned to the two lowest triage levels on a 5-level scale (validity).

## Introduction

Triage is a central task in an emergency department (ED). In this context, triage is viewed as the rating of patients' clinical urgency [1]. Rating is necessary to identify the order in which patients should be given care in an ED when demand is high. Triage is not needed if there is no queue for care. Triage scales aim to optimize the waiting time of patients according to the severity of their medical condition, in order to treat as fast as necessary the most intense symptom(s) and to reduce the negative impact on the prognosis of a prolonged delay before treatment. ED triage is a relatively modern phenomenon, introduced in the 1950s in the United States [2]. Triage is a complex decision-making process, and several triage scales have been designed as decision-support systems [3] to guide the triage nurse to a correct decision. Triage decisions may be based on both the patients' vital signs (respiratory rate, oxygen saturation in blood, heart rate, blood pressure, level of consciousness, and body temperature) and their chief complaints. Internationally, no consensus has been reached on the functions that should be measured. Apart from emergency care, triage may be used in other clinical activities, e.g. deciding on a certain investigation [4] or treatment [5].

Since the early 1990s, several countries have developed and introduced ED triage [6-10]. Development of triage scales in some countries has been influenced largely by the seminal work of FitzGerald [11], resulting in most of the triage scales developed in the 1990s and 2000s being designed as 5-level scales. Of these, the Australian Triage Scale (ATS), Canadian Emergency Department Triage and Acuity Scale (CTAS), Manchester Triage Scale (MTS), and Emergency Severity Index (ESI) have had the greatest influence on modern ED triage [12-15]. Other scales have not disseminated as widely around the globe, e.g. the Soterion Rapid Triage Scale (SRTS) from the United States and the 4-level Taiwan Triage System (TTS) [6,7,9,16,17]. Some countries, e.g. Australia, have a national mandatory triage scale while many European countries lack such standards [7,9].

Patients may have a life-threatening condition, but show normal vital signs. Hence, in triaging the patient it is important to consider information given by patients or accompanying persons regarding the patient's chief complaints or medical history, which can provide essential information about serious diseases. The chief complaints describe the incident or symptoms that caused the patient to seek care.

In 2005, a joint task force of the American College of Emergency Physicians and the Emergency Nurses Association published a review of the literature on ED triage scales. Based on expert consensus and available evidence, the task force supported adoption of a reliable 5-level triage scale, stating that either the CTAS or the ESI are good choices for ED triage [18]. In 2002, a national survey conducted in Sweden identified the use of 37 different triage scales across the country. Further, some 30 EDs did not use any type of triage scale [19].

This systematic review aims to investigate the scientific evidence underlying published ED triage scales.

## Objectives

The following questions are addressed:

1. In triage of adults at EDs, does assessment of individual vital signs or chief complaints affect mortality during the hospital stay or within 30 days after arrival at the ED?

2. In adult ED patients, what is the level of agreement between clinicians' triage decisions compared to each other or to a gold standard for each scale (i.e. the reliability of triage scales)?

3. In adult ED patients, how valid is each triage scale in predicting hospitalization and hospital mortality?

## Methods

A systematic search of the international literature published from 1966 through March 31, 2009 explored the British Nursing Index, Business Source Premier, CINAHL, Cochrane Library, EMBASE, and PubMed. Inclusion was limited to studies of adult patients (≥15 years) visiting EDs for somatic reasons. Another criterion for inclusion was that the study design must contain a control, i.e. randomized controlled trials (RCT), observational studies with a control group based on previously collected data, and before-after studies. Descriptive studies without a control group and retrospective studies were excluded.

### Inclusion criteria for vital signs and chief complaints used in triage scales

• Studies analyzing individual vital signs or chief complaints

• Outcome variable defined as death within 30 days after ED arrival or during the hospital stay

### Inclusion criteria for reliability and validity of triage scales

• Studies based on real patients triaged at EDs (validity)

• Studies based on real patients triaged at EDs or fictitious patient scenarios (reliability)

• Studies reporting reliability at separate triage levels (reliability)

• Studies reporting mortality and hospitalization per triage level (validity)

• Outcome variables defined as death in the ED or hospital, and need for hospitalization (validity)

### Exclusion criteria for studies on reliability of triage scales

• Studies on interrater reproducibility are excluded in cases where any rater in the study had access to retrospective data only.

Six experts from different professions and clinical specialties reviewed the studies, independently in groups of 2 or 3, for quality by using methods validated for internal validity, precision, and applicability (external validity) [20]. The *methodological quality and clinical relevance *of each study was graded as high, medium, or low. Results from the studies that met the inclusion criteria and quality standards were synthesized by applying the internationally developed GRADE system [21].

In accordance with GRADE, the following factors were considered in appraising the overall strength of the evidence: study quality, concordance/consistency, transferability/relevance, precision of data, risk of publication bias, effect size, and dose-response. In synthesizing the data, studies having *low *quality and relevance were included when studies of *medium *quality and relevance were not available. Based on the overall quality and relevance of the studies reviewed, each conclusion was rated as having strong, moderately strong, limited, or insufficient scientific evidence. If studies were not available, this was noted [21].

## Results

**Figure 1:**
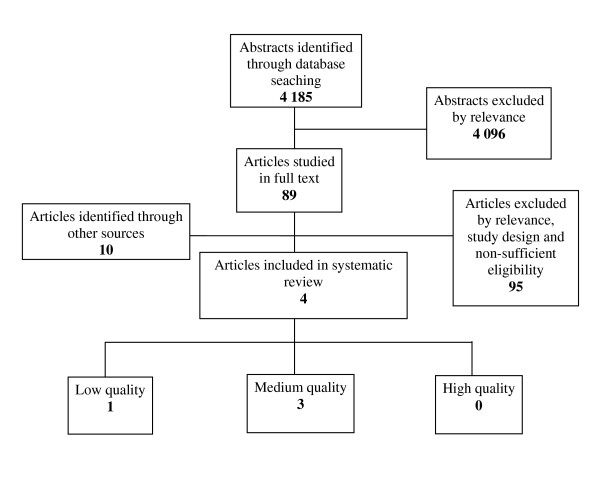
**Results of literature search and selection process**.

**Figure 2:**
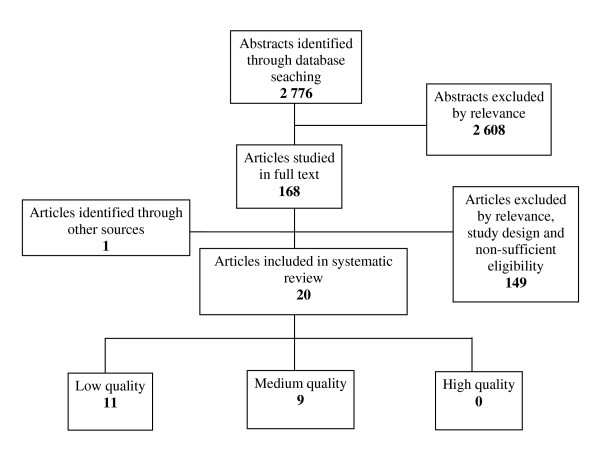
**Results of literature search and selection process regarding reliability (10 articles), and validity (10 articles) of triage scales**. *One article studied both reliability and validity and was rated differently due to the studied endpoint, low quality regarding reliability and medium quality regarding validity*.

Figures 1 and 2 illustrate the results of the primary search.

### Vital signs and chief complaints

Most of the studies that investigated associations between different vital signs or chief complaints and mortality after ED arrival were observational cohort studies based on selected, diagnosis-specific, patient groups. All of the studies were found to have medium quality and relevance. Only a few studies included all patients (albeit limited to "medical" patients") that arrived at the ED, regardless of diagnosis. Hence, studies of patients classified as surgical disciplines were generally lacking. Several studies described compiled scales or indexes for appraising the severity level of the patient's conditions, but provided no information on the importance of specific vital signs or chief complaints. Hence, little or no evidence can be found on the association between specific vital signs or reasons for the ED visit and mortality in the group of general patients presenting in EDs.

#### Respiratory rate

Only a single study, which described the predictive importance of respiratory rate, fulfilled the inclusion criteria [22]. The study aimed to assess whether the Rapid Acute Physiology Score (RAPS) could be used to predict mortality in nonsurgical patients on ED arrival. It also aimed to study whether an advanced version of RAPS, i.e. the Rapid Emergency Medicine Score (REMS), could yield better predictive information [22].

RAPS was developed for prehospital care and involves assessing respiratory rate, pulse, blood pressure, and the Glasgow Coma Scale (GCS). REMS is based on RAPS, but also assesses oxygen saturation, body temperature, and age. In total, 11 751 patients were studied prospectively after arrival at the ED of a university hospital in Sweden. Respiratory rate was found to be a significant predictor of mortality during the hospital stay. A decrease of one step on the RAPS scale was found to nearly double the risk of mortality within 30 days (Table 1).

**Table 1:** Does assessment of certain vital signs and chief complaints in emergency department triage of adults have an impact on 30-day or in-hospital mortality?

Author Year, reference Country	Study design	Patient characteristics Sample Female/age Male/age Inclusion criteria Type of emergency department	Primary outcome	Outcome Frequency RR (relative risk), OR (odds ratio) P-value, 95% CI (confidence interval)	Missing data (%)	Study quality and relevance Comments
Goodacre S et al 2006 [23] United Kingdom	Observational Cohort Retrospective database review	Emergency medical admissions, life threatening category A emergency calls N = 5 583 Female: 2 350 (42.3%) Male: 3 233 (57.7%) Mean age 63.4 years **Inclusion criteria: **Any case where caller report chest pain, unconsciousness, not breathing and patient admitted to hospital or died in emergency department (ED) **Setting: **variables recorded on ambulance arrival	Mortality in hospital during the stay	Age, Glascow Coma Scale (GCS) and oxygen saturation independent predictors of mortality in multivariate analysis, blood pressure is not useful Glascow Coma Scale (GCS): OR 2.10 (95% CI 1.86-2.38) p < 0.001 **Age**: OR 1.74 (95% CI 1.52-1.98) p < 0.001 **Saturation**: OR 1.36 (95% CI 1.13-1.64) p = 0.001	Rapid Acute Physiology Score (RAPS - blood pressure, pulse, GCS, RR, saturation and temp) in only 3 624 (64.9%). Missing in 35.1% Rapid Emergency Medicine Score (REMS - Blood pressure, pulse, GCS, RR) in only 2 215 (39,7%). Missing in 60.3%. New Score (GCS, saturation, age) in 2 743 (49.1%). Missing in 50.9%	Moderate Acceptable external validity Good/acceptable internal validity Age, GCS and saturation independent predictors of mortality. Blood pressure is not a useful predictor

Olsson T et al 2004 [22] Sweden	Observational cohort Prospective	Nonsurgical emergency department (ED) patients n = 11 751 Female: 51.6% Male: 48.4% Mean age 61.9 (SD ± 20.7) **Inclusion criteria: **Patients consecutively admitted to the emergency department (ED) over 12 months. **Exclusion criteria: **Patients with cardiac arrest that could not be resuscitated, patients with more than one parameter missing. **Setting: **1 200 bed University hospital ED in Sweden	Mortality in hospital, within 48 hours	In-hospital mortality 2.4%, mortality within 48 hours 1.0%. Predictors for mortality: **Saturation **OR: 1.70 (95% CI: 1.36-2.11) p < 0.0001 **Respiratory frequency **OR: 1.93 (95% CI: 1.37-2.72) p < 0.0002 **Pulse frequency **OR 1.67 (95% CI 1.36-2.07) p < 0.0002 **Coma **OR: 1.68 (95% CI: 1.38-2.06) p < 0.0001 **Age **OR: 1.34 (95% CI: 1.10-1.63) p < 0.004		Moderate Good internal validity

Han JH et al 2007 [25] USA Singapore	Observational cohort Retrospective database review Comparison patients ≥/≤ 75 years	Suspected acute coronary syndrome (ACS) n = 10 126 Female: 5 635 Male: 4 491 Mean age = ? 11.4% ≥75 years **Inclusion criteria: **≥ age 18, suspected ACS verified by electrocardiogram (ECG), cardiac biomarkers, dyspnoea, light-headedness, dizziness and weakness. **Exklusion criteria: **Inter-hospital transfer, if missing data concerning gender, age or clinical presentation **Setting: **8 emergency departments (ED) (USA), 1 ED (Singapore)	Mortality in-hospital/within 30 days	2.7% in-hospital mortality for patients **age **≥75 years, higher 30 day mortality (Adjusted OR: 2.6, 95% CI: 1.6-4.3)	Missing data for ECG, symptoms or gender in 1 810 (15.2%)	Low Convenience sample-selection bias Confounders, such as co-morbidity not described Acceptable intern validity

Arboix A et al 1996 [24] Spain	Observational cohort	Stroke n = 986 Female: 468 Male: 518 Mean age = ? **Inclusion criteria: **First-ever stroke, admitted to hospital. **Setting: **Department of neurology, university hospital	Mortality in-hospital	Overall mortality 16.3%. **Age **OR: 1.05 (95% CI: 1.03-1.07), previous or concomitant **Pathologic conditions **OR: 1.83 (95% CI: 1.19-2.82) Deteriorated level of **Consciousness **OR: 11.70 (95% CI: 7.70-17.77) **Vomiting **OR: 2.18 (95% CI: 1.20-3.94) **Cranial nerve palsy **OR: 2.61 (95% CI: 1.34-5.09) **Seizures **OR: 5.18 (95% CI: 1.70-15.77) and **Limb weakness **OR: 3.79 (95% CI: 1.96-7.32) were independent prognostic factors of in-hospital mortality	Not stated	Moderate

#### Oxygen saturation in blood

Two studies used RAPS and REMS to predict acute mortality after ED arrival and specifically studied the predictive importance of saturation [22,23]. Oxygen saturation was found to be one of the three variables, along with age and level of consciousness, that best predicted mortality during hospitalization.

#### Pulse

One study investigated the importance of assessing pulse in the ED as a means to predict mortality during the hospital stay.

The study, which was conducted in Sweden [22], showed a significant association between the pulse on arrival to the ED and mortality during the hospital stay in a group of 11 751 patients receiving care for nonsurgical disorders. With a decrease of one step on the RAPS scale, 67% of the patients showed an increased risk of mortality within 30 days.

#### Level of consciousness

The Swedish study (described above) also investigated the association between acute mortality and the level of consciousness on arrival at the ED [22]. Another study used the same methods mentioned above, i.e. RAPS and REMS [23], to analyze 5583 patients that had called the emergency phone number and were classified as urgent. The study showed that level of consciousness was one of three variables (age and saturation being the other two) that best predicted mortality during the hospital stay. Another study analyzed 986 stroke patients on ED arrival. Impaired level of consciousness appeared to be the best predictor of mortality during the hospital stay [24].

#### Blood pressure and body temperature

The importance of blood pressure or body temperature in assessing the risk of acute mortality after ED arrival could not be supported by the included studies due to the lack of scientific evidence.

#### Chief complaints

Studies describing the association between different chief complaints and acute mortality were found to be lacking.

#### Age

Three of the studies described above showed that the higher the patient's age, the greater the risk of death within 30 days of hospital care following ED arrival [22-24]. The results showed an increase in mortality of 5% per year. Furthermore, one study showed that older patients (above 75 years of age) with symptoms of coronary heart disease had a greater risk of death within 30 days after arrival at the ED compared to younger patients with the same symptoms [25] (Table 1).

Based on the studies described above, Table 2 summarizes assessments and comments regarding the level of scientific evidence.

**Table 2:** Appraisal of scientific evidence according to GRADE - Association between vital signs/chief complaints and acute mortality after arrival at the emergency department.

Effect measure (endpoint)	No. Patients (no. Studies) Reference	Effect (OR, odds ratio*)	Scientific evidence	Comments
Respiratory rate predicts 30-day mortality	11 751 1 study [22]	1.9	Insufficient ⊕○○○	Only one study (-1)

Oxygen saturation predicts 48-hour mortality or in-hospital mortality	17 334 2 studies [22,23]	1.4 1.7	Limited ⊕⊕○○	

Pulse predicts 30-day mortality	11 751 1 study [22]	1.7	Insufficient ⊕○○○	Only one study (-1)

Level of consciousness predicts 48-hour mortality or in-hospital mortality	18 320 3 studies [22-24]	2.1 1.7 11.7	Limited ⊕⊕○○	

Age predicts 30-day mortality	28 446 4 studies [22-25]	1.7 1.3 2.6 1.1	Moderate ⊕⊕⊕○	Upgrading due to effect size and dose-response effect (+1)

All studies are observational.

* OR indicates each step of change in RAPS (Rapid Acute Physiology Score) or REMS (Rapid Emergency Medicine Score).

### Interrater agreement of triage scales (reliability)

All 11 articles that were found to answer the question concerning reliability of triage scales and met the defined inclusion criteria were observational studies. They addressed reliability of the ATS [26], CTAS (including eTriage) [19,27-30], MTS [31], SRTS [6], and two locally produced scales without names [8,32] (Table 3). Based on the quality review, 9 articles [6,8,19,26-31] were found to be of low and 1 [32] of medium quality. One article was excluded due to deficient quality resulting from high internal dropout [16]. Deficient external validity was the major reason for the low- and medium-quality ratings of the studies. Selection of patients and triage nurses were both found to be irrelevant or insufficiently described. Hence, 10 articles remained as a basis for the conclusions.

**Table 3:** Reliability of triage scales

Author Year, reference Country	Triage system	Patient characteristics: Age Gender Triageur: Amount, profession	Results: κ-values, percentage agreement (PA)/triage level	Drop out (%)	Study quality and relevance
Considine J et al 2000, [26] Australia	ATS	10 scenarios 31 RNs	Triage level: 1: 59.7% PA 2: 58% PA 3: 79% PA 4: 54.8% PA 5: 38.7% PA	0%	Low External validity is uncertain, internal validity is good while sample size is of uncertain adequacy

Dong S et al 2006, [28] Canada	ETriage (CTAS)	569 patients 49.4 years 51 % male Unknown amount of RNs	0.40 (unweighted κ) Triage level: 1: 62.5% PA 2: 49.5% PA 3: 59.7% PA 4: 68.5% PA 5: 43.5% PA	1%	Low External validity can not be assessed, internal validity is excellent while sample size is of uncertain adequacy

Dong S et al 2005, [29] Canada	CTAS/eTriage	693 patients 48 years 49 % male 73 RNs	0.202 (unweighted κ) Triage level: 1: 50% PA 2: 9% PA 3: 53.5% PA 4: 73.3% PA 5: 7.2% PA	4%	Low External validity can not be assessed, internal validity is excellent while sample size is of uncertain adequacy

Manos D et al 2002, [30] Canada	CTAS	42 scenarios 5 BLS 5 ALS 5 RNs 5 Drs	0.77 overall (weighted κ) BLS: 0.76 (weighted κ) ALS: 0.73 (weighted κ) RNs: 0.80 (weighted κ) Drs: 0.82 (weighted κ) Triage level: 1: 78% PA 2: 49% PA 3: 37% PA 4: 41% PA 5: 49% PA	0.2%	Low External validity can not be assessed, internal validity is acceptable while sample size is of uncertain adequacy

Beveridge R et al 1999, [27] Canada	CTAS	50 scenarios 10 RNs 10 Drs	0.80 overall (weighted κ) 0.84 RNs (weighted κ) 0.83 Drs (weighted κ) Weighted κ / triage level (RNs): Triage level: 1: 0.73 2: 0.52 3: 0.57 4: 0.55 5: 0.66	15%	Low External validity can not be assessed, internal validity is acceptable while sample size is of uncertain adequacy

Göransson K et al 2005, [19] Sweden	CTAS	18 scenarios 423 RNs	0.46 (unweighted κ) Triage level: 1: 85.4% PA 2: 39.5% PA 3: 34.9% PA 4: 32.1% PA 5: 65.1% PA	0.8%	Low External validity can not be assessed, internal validity is acceptable while sample size is of uncertain adequacy

van der Wulp I et al 2008, [31] The Netherlands	MTS	50 scenarios 55 RNs	0.48 (unweighted κ) Triage level: 2: 9.8% PA 3: 35.5% PA 4: 22% PA	7.5-35.7%	Low External validity is uncertain, internal validity is good while sample size is of uncertain adequacy

Maningas P et al 2006, [6] USA	SRTS	423 patients 29.7 years 44% male 16 RN pairs	0.87 (weighted κ) Triage level: 1: 85.7% PA 2: 86.7% PA 3: 86.8% PA 4: 93.9% PA 5: 74.2% PA		Low External validity can not be assessed, internal validity is good while sample size is of uncertain adequacy

Rutschmann OT et al 2006, [8] Switzerland	4-tier system	22 patient scenarios 45 RNs 8 Drs	RNs: 0.40 (weighted κ) Drs: 0.28 (weighted κ) Triage level: 1: 61% PA 2: 49.6% PA 3: 74.2% PA 4: 75.5% PA	4% 0%	Low External validity is uncertain, internal validity is excellent while sample size is of uncertain adequacy

Brillman J et al 1996, [32] USA	4-tier system	5 123 patients 64% < 35 years 54% male Unknown amount of RNs and Drs	0.45 (unknown type of κ) Triage level: 1: 0.13% PA 2: 5.2% PA 3: 37.9% PA 4: 24.6% PA	10%	Moderate External validity is clear, internal validity is good while sample size is of uncertain adequacy

ATS = Australasian Triage Scale; CTAS = Canadian Emergency Department Triage and Acuity Scale; MTS = Manchester Triage Scale; SRTS = Soterion Rapid Triage Scale; RNs = registered nurses; Drs = doctors; BLS = Basic Life Support; ALS = Advanced Life Support

The scientific evidence was found to be insufficient to assess the reliability of ATS, CTAS, MTS, SRTS and the Swiss scale (Table 4). However, limited scientific evidence was found in assessing the reproducibility of the Brillman scale (North America) as having moderate interrater agreement.

**Table 4:** Appraisal of scientific evidence (according to GRADE) - Reliability of triage scales.

Effect measure (endpoint)	Triage scale	No. Patients/cases (no. Studies)	Agreement (Kappa/ percent)	Scientific evidence	Comments
Reliability	ATS	10 cases (1 study) [26]	38.7%-79%	Insufficient ⊕○○○	Reduction for study quality and imprecise data (-1)

	CTAS	1372 patients/cases (5 studies) [19,27-30]	0.20-0.84 (κ-value)	Insufficient ⊕○○○	Reduction for study quality and heterogeneity of results (-1)

	MTS	50 cases (1 study) [31]	0.48 (κ-value)	Insufficient ⊕○○○	Reduction for study quality and imprecise data (-1)

	SRTS	423 patients (1 study) [6]	0.87 (κ-value)	Insufficient ⊕○○○	Reduction for study quality and uncertainty of transferability (-1)

	Rutschmann	22 cases (1 study) [8]	0.28-0.40 (κ-value)	Insufficient ⊕○○○	Reduction for study quality (-1)

	Brillman	5123 patients (1 study) [32]	0.45 (κ-value)	Limited ⊕⊕○○	

All studies are observational.

### Validity of triage scales regarding acute mortality and hospital admission rates

#### Mortality

None of the studies reported on hospital admission rates adjusted for age and gender or mortality (Table 5). Since previous studies have shown that age is one of the major predictors of hospital mortality [33,34] the scientific evidence was found to be insufficient to asses the validity of the triage scales ATS, CTAS, and Medical Emergency Triage and Treatment System (METTS) (Table 6). However, safety as measured by hospital mortality in patients graded as low risk (triage levels 4-5/green-blue) by the triage systems may be regarded as one aspect of validity. When assessing the above-mentioned triage scales' level of validity as regards mortality at the lowest triage levels *only *(levels 4-5/green-blue), the quality and relevance of the studies were found to be moderate. Hence, scientific evidence is limited.

**Table 5:** Studies on how the assessment of the urgency of need to see a physician according to different triage systems could predict hospital mortality.

Author Year, reference Country	Triage system	Patient characteristics: Age Gender	Outcome	Results (Mortality frequency per triage level)	Remarks	Study quality and relevance
Dong SL et al 2007, [43] Canada	ECTAS	29 346 patients 47 years 48% female	Mortality in ED	Triage level: 1: 22% 2: 0.22% 3: 0.031% 4: 0.018% 5: 0% OR 664 (357-1233), 1 vs 2-5	- Low number of fatalities (70 cases)	Moderate

Dent A et al 1999, [35]	ATS	42 778 patients Age & sex not given	In-hospital mortality	Triage level: 1: 16% 2: 5% 3: 2% 4: 1% 5: 0.1% p < 0.0001		Moderate

Widgren BR et al 2008, [10] Sweden	METTS	8 695 patients 65 years 45% female	In-hospital mortality	Triage level: 1: 14% 2: 6% 3: 3% 4: 3% 5: 0.5% p < 0.001	- Only patients admitted to hospital evaluated	Moderate

Doherty SR et al 2003, [36]	ATS	84 802 patients Age & sex not given	24 hours mortality	Triage level: 1: 12% 2: 2.1% 3: 1.0% 4. 0.3% 5: 0.03% p < 0.001	- Consecutive patients	Moderate

Mortality figures (%) are shown for each triage level for patients admitted to a hospital emergency department.

CTAS = Canadian Emergency Department Triage and Acuity Scale; ATS = Australian Triage Scale; METTS = Medical Emergency Triage and Treatment System

**Table 6:** Appraisal of scientific evidence (according to GRADE) - Validity of 5-level triage scales measured by acute mortality.

Effect measure (endpoint)	Triage scale	No. Patients (no. Studies)	Mortality at triage level 5 (percent)	Scientific evidence	Comments
Patient mortality	CTAS	29 346 (1 study) [43]	0%	Limited ⊕⊕○○	Only one study, but large population

	ATS	127 079 (2 studies) [35,36]	0.03%-0.1%	Limited ⊕⊕○○	

	METTS	8695 (1 study) [10]	0.5%	Insufficient ⊕○○○	Reduction for study quality (-1)

All the studies are observational

#### Hospital admission rates in patients triaged as non-acute

Nine studies reported on admission rates for the ESI, ATS, and SRTS triage scales (Table 7). The studies showed a range between 0.0% and 17.0% at level 5, the lowest triage level [6,16,35-41]. A range was also observed in the age panorama (mean ages between 30 and 47 years) and in hospital admission rates at triage level 4 (3%-33%): 18% to 33% for ATS, 6% to 10% for ESI, and 3% for SRTS.

**Table 7:** Studies on how the assessment of the urgency of need to see a physician according to different triage systems could predict hospitalization.

Author Year, reference Country	Triage system	Patient characteristics: Age Gender	Outcome	Results (Hospital admission frequency per triage level)	Comments	Study quality and relevance:
Van Gerven R et al 2001, [39] The Netherlands	ATS	3 650 patients, Age & sex not given	Hospital admission	Triage level: 1: 85% 2: 71% 3: 48% 4: 18% 5: 17% p < 0.0001		Moderate

Chi CH et al 2006, [16] Taiwan	ESI2	3 172 patients 47 years 47% female	Hospital admission	Triage level: 1: 96% 2: 47% 3: 31% 4: 7% 5: 7% p < 0.0001	- ESI scored in retrospect - Unclear inclusion criteria	Moderate

Wuerz RC et al 2000, [40] USA	ESI	493 patients 40 years 52% female	Hospital admission	Triage level: 1: 92% 2: 61% 3: 36% 4: 10% 5: 0 % p < 0.0001	- Unclear inclusion criteria	Low

Dent A et al 1999, [35]	ATS	42 778 patients Age & sex not given	Hospital admission	Triage level: 1: 83% 2: 69% 3: 49% 4: 33% 5: 9% p < 0.0001		Moderate

Eitel DR et al 2003, [37] USA	ESI2	1 042 patients 7 different EDs 43 years 47% female	Hospital admission	Triage level: 1: 83% 2: 67% 3: 42% 4: 8% 5: 4% p < 0.001	- Not consecutive patients	Moderate

Tanabe P et al 2004, [38] USA	ESI3	403 patients 45 years 49% female	Hospital admission	Triage level: 1: 80% 2: 73% 3: 51% 4: 6% 5: 5% p < 0.001	- Not consecutive patients - Retrospective triage	Low

Wuerz RC et al 2001b, [41] USA	ESI	8 251 patients Age & sex not given	Hospital admission	Triage level: 1: 92% 2: 65% 3: 35% 4: 6% 5: 2% p < 0.001	- consecutive patients	Moderate

Doherty S et al 2003, [36]	ATS	84 802 patients Age & sex not given	Hospital admission	Triage level: 1: 79% 2: 60% 3: 41% 4: 18% 5: 3.1% p < 0.001	- consecutive patients	Moderate

Maningas PA et al 2006, [6]	SRTS	33 850 patients Age 30, 56% female	Hospital admission	Triage level: 1: 43% 2: 30% 3: 13% 4: 3.0% 5: 1.4% p < 0.0001	- consecutive patients	Moderate

Hospitalization figures (%) are shown for each triage level for patients admitted to a hospital emergency department.

ATS = Australian Triage Scale; ESI = Emergency Severity Index; SRTS = Soterion Rapid Triage Scale.

Seven of these studies were found to be of moderate and two of low quality and relevance, and the scientific evidence for validity of admission rates for patients in the lowest triage levels (levels 4-5/green-blue) was found to be limited (Table 8).

**Table 8:** Appraisal of scientific evidence (according to GRADE) - Safety of 5-level triage scales as measured by hospitalisation rates in patients at triage level 5.

Effect measure (endpoint)	Triage scale	No. patients (no. studies)	Hospitalization rate at triage level 5 (percent)	Scientific evidence	Comments
Patient safety related to hospital admission	ATS	131 230 (3 studies) [35,36,39]	3.1%-17%	Limited ⊕⊕○○	

	ESI	13 361 (5 studies) [16,37,38,40,41]	0%-7%	Limited ⊕⊕○○	

	SRTS	33 850 (1 study) [6]	1.4%	Limited ⊕⊕○○	Only one study, but many patients

All studies are observational.

## Discussion

Our systematic review shows that when adjudicated by standard criteria for study quality and scientific evidence, the triage scales used in EDs are supported, at best, by *limited evidence*. Often, the evidence is weaker, not above *insufficient *by the GRADE criteria. The ability of the individual vital signs included in the different scales to predict outcome has seldom, or never, been studied in the ED setting. The scientific evidence for assessing interrater agreement (reproducibility) was limited for one triage scale (Brillman) whereas it was insufficient or lacking for all other scales. Two of the scales (CTAS and ATS) offered limited scientific evidence, and the scientific evidence for one scale (METTS) was insufficient to assess the risk of early death or hospitalization in patients assigned to the two lowest triage levels in 5-level scales; the studies showed the risk of death to be low, but a need for inpatient care was not excluded (about 5% hospital admission rate on average). Studies on validity of the triage scales across all levels, i.e. their ability to distinguish the urgency in patients assigned the five different levels, were generally of low quality. Consequently, evidence was insufficient to assess the validity of the scales.

As none of the studies reported on mortality rates adjusted for differences in age and gender between the triage levels, we could not evaluate the validity of the triage scales across all triage levels as regards the risk of early death. To estimate the safety of the scales, we studied early death among patients assigned to the lowest triage levels (green and blue/4-5). Two triage scales (ATS and CTAS) offered limited scientific evidence for assessing safety. In both scales, the patients assigned to the two lowest triage levels had a very low risk of dying within 24 hours after triage. Hence, in this respect, the scales are safe to use. Scientific evidence for METTS, the newly developed Swedish triage scale, was found to be insufficient to assess safety. Since the study recorded the risk of dying during the in-hospital stay, mortality was higher than in the studies on ATS and CTAS.

In using the need of hospitalization as a measure of safety, the situation was found to be more complex. Again, none of the studies reported on hospital admission rates adjusted for age and gender, so we could not evaluate the validity of the triage scales across all triage levels. However, on average, about 5% (in some studies up to 17%) of patients in the lowest (4-5/green-blue) triage levels in ATS, ESI, and SRTS were reported to be admitted as inpatients. The variations were wide not only between different triage scales, but also between studies using the same scales. This indicates differences between the studies in (a) patient populations in the ED, (b) access to hospital beds, (c) hospital admission policies and traditions, and/or (d) inaccurate triage decisions (i.e. patients were rated as less urgent than their actual urgency).

No definitive conclusions could be drawn regarding which of the scales was the safest as measured by the need of hospitalization. Hence, we suggest that none of the scales be used in referral of patients in the lowest triage levels (4-5/green-blue), e.g. to primary care, without further medical examination in the ED.

New diagnostic tests typically need to meet rigid criteria before they can be accepted for widespread use. These criteria include documentation on precision. For non-laboratory tests, interrater agreement (reliability) is a key precision issue. Our review shows that most triage scales present insufficient scientific evidence for assessing interrater agreement. The study designs used to estimate interrater agreement have often been suboptimal. Most of the studies are based on fictitious cases rather than on authentic patients in real-life settings. The value of the studies as regards interrater agreement is also compromised by the fact that the mean age of patients assessed has either been low (as low as 30 years) or unreported. The generalizability to real-life ED patients must therefore be questioned.

All 5-level triage scales present insufficient evidence on interrater variability. The few studies that have been published (most of low quality) have reported widely divergent interrater agreement, with kappa values ranging from 0.2 (slight agreement) to 0.9 (almost perfect). Only a single study [32] presented limited scientific evidence. This was a 4-grade scale reporting a kappa value of 0.45, a value usually considered to be in the moderate agreement range [42]. It is evident that inter-observer agreement in triage scales must be documented in greater detail, and, if low, actions must be taken to reduce variability.

The literature shows variations in the vital signs and chief complaints applied in triage scales. It is unclear whether the selected vital signs are the best at distinguishing different risk groups. Further, evidence supporting the selected thresholds for continuous variables is deficient. The inclusion criteria for this systematic literature review place considerable emphasis on relevance. Triage scales are intended to be used in EDs irrespective of specific symptoms or disease. Hence, only studies of unselected patient populations in ED settings were included, greatly limiting the number of studies on the ability of individual vital signs to predict outcome. Our literature search revealed that many more studies had been performed in intensive care units, or soon after hospital admission.

Regarding specific vital signs, limited scientific evidence supports the use of oxygen saturation and consciousness level as predictors of mortality early after triage. However, scientific evidence was found to be insufficient as regards respiration and pulse, blood pressure, and body temperature. Hence, it remains unclear whether the selected vital signs are the best ones to use in distinguishing different risk groups. Moderate scientific evidence indicated age as a predictor of mortality early after triage, yet most triage scales do not take age into account.

MTS and eCTAS include the chief complaint leading to the ED visit, but we did not find any studies that analyzed which of the chief complaints are important predictors of mortality early after triage. It appears likely that in the construction of triage scales, much of the information was deduced from studies performed in settings other than EDs.

### Strengths and limitations

The strength of this review of the scientific literature on triage in the ED lies in its systematic approach. Our search for relevant literature has been meticulous; the quality of the included studies has been evaluated in a uniform manner; and the level of evidence has been summarized using the GRADE methodology developed under the auspices of the World Health Organization [21].

Our review is limited to ED triage in adult patients in somatic care. However, EDs are only part of a continuum of services for acutely ill and injured patients. Studies are also needed in other aspects along the continuum of care, e.g. prehospital, psychiatric, and pediatric triage. Other limitations are ascribed to the volume and quality of the scientific literature available. Since all studies were observational, none of the evidence came from randomized controlled trials, the "gold standard" for evaluating new methods. As none of the studies met the standards for high quality, we included studies of low and moderate quality in our review in accordance with the creed in evidence based medicine to use the *best available *scientific evidence. Low study quality affected the GRADE rating and was a reason why scientific evidence was rated as insufficient or limited for so many aspects of so many scales.

## Conclusions

This systematic literature review reveals shortcomings in the scientific evidence on which presently available triage scales are based. Stronger scientific evidence is needed to determine which of the vital signs and chief complaints have the greatest prognostic value in triage. Interrater agreement (reliability), validity, and safety of triage scales need to be investigated further, and head-to-head comparisons are needed to determine whether any of the scales have advantages over others.

## Limitations

This review was confined to ED triage scales for adult ED patients with non-psychiatric illnesses or injuries. In the absence of an internationally agreed outcome measure for ED triage scale validity, the proxy variables hospital admission and mortality were used in the current study. These proxy variables have limitations with regards to ED triage scale validity as the variables may be affected by events occurring after the triage assessment. Further, comparison between ED triage scales need to be done with caution as there may be contextual differences influencing the result.

## Competing interests

The authors declare that they have no competing interests.

## Authors' contributions

All authors contributed to study concept and design, and acquisition, analysis, and interpretation of the data. Finally all authors read and approved the submitted manuscript.
